# Expert consensus on the treatment of oral diseases in pregnant women and infants

**DOI:** 10.1038/s41368-025-00395-3

**Published:** 2025-08-29

**Authors:** Jun Zhang, Chenchen Zhou, Liwei Zheng, Jun Wang, Bin Xia, Wei Zhao, Xi Wei, Zhengwei Huang, Xu Chen, Shaohua Ge, Fuhua Yan, Jian Zhou, Kun Xuan, Li-An Wu, Zhengguo Cao, Guohua Yuan, Jin Zhao, Zhu Chen, Lei Zhang, Yong You, Jing Zou, Weihua Guo

**Affiliations:** 1 Yunnan Key Laboratory of Stomatology & Department of Pediatric Dentistry, The Affiliated Stomatology Hospital, Kunming Medical University, Kunming, China; 2 State Key Laboratory of Oral Diseases & National Clinical Research Centre for Oral Diseases & Department of Pediatric Dentistry, West China Hospital of Stomatology, Sichuan University, Chengdu, China; 3 Department of Pediatric Dentistry, Ninth People’s Hospital, School of Medicine, Shanghai Jiao Tong University, Shanghai Key Laboratory of Stomatology, Shanghai, China; 4 Department of Pediatric Dentistry, Peking University School and Hospital of Stomatology, Beijing, China; 5 Department of Pediatric Dentistry, Guanghua School of Stomatology, Guangdong Provincial Key Laboratory of Stomatology, Sun Yat-Sen University, Guangzhou, China; 6 Hospital of Stomatology, Guanghua School of Stomatology, Sun Yat-Sen University, Guangdong Provincial Key Laboratory of Stomatology, Guangzhou, China; 7 Department of Endodontics, Shanghai Ninth People’s Hospital, College of Stomatology, Shanghai Jiao Tong University School of Medicine, National Clinical Research Center for Oral Diseases, National Center for Stomatology, Shanghai Key Laboratory of Stomatology, Shanghai, China; 8 Department of Pediatric Dentistry, School and Hospital of Stomatology, China Medical University, Shenyang, China; 9 Department of Periodontology, School and Hospital of Stomatology, Cheeloo College of Medicine, Shandong University & Shandong Key Laboratory of Oral Tissue Regeneration, Shandong Engineering Research Center of Dental Materials and Oral Tissue Regeneration, Shandong Provincial Clinical Research Center for Oral Diseases, Jinan, China; 10 Department of Periodontology,Nanjing Stomatological Hospital, Affiliated Hospital of Medical School, Institute of Stomatology, Nanjing University, Nanjing, China; 11 Department of VIP Dental Service, Beijing Key Laboratory of Tooth Regeneration and Function Reconstruction, School of Stomatology, Capital Medical University, Beijing, China; 12 Department of Pediatric Dentistry, School of Stomatology, State Key Laboratory of Military Stomatology, National Clinical Research Center for Oral Diseases, Shanxi Key Laboratory of Military Stomatology, Fourth Military Medical University, Xi’an, China; 13 State Key Laboratory of Oral & Maxillofacial Reconstruction and Regeneration, Key Laboratory of Oral Biomedicine Ministry of Education, Hubei Key Laboratory of Stomatology, Department of Periodontology, School & Hospital of Stomatology, Wuhan University, Wuhan, China; 14 Department of Cariology and Endodontics, The First Affiliated Hospital of Xinjiang Medical University (The Affiliated Stomatology Hospital of Xinjiang Medical University), Urumqi, China; 15 School of Stomatology, Zunyi Medical University, Zunyi, China. Department of Endodontics, Guiyang Stomatological Hospital, Guiyang, China; 16 Hohhot Stomatology Hospital (Inner Mongolia Autonomous Stomatology Hospital), Hohhot, China; 17 Department of Obstetrics and Gynecology, West China Second University Hospital, Sichuan University, Chengdu, China

**Keywords:** Paediatric dentistry, Dental diseases

## Abstract

With the growing emphasis on maternal and child oral health, the significance of managing oral health across preconception, pregnancy, and infancy stages has become increasingly apparent. Oral health challenges extend beyond affecting maternal well-being, exerting profound influences on fetal and neonatal oral development as well as immune system maturation. This expert consensus paper, developed using a modified Delphi method, reviews current research and provides recommendations on maternal and child oral health management. It underscores the critical role of comprehensive oral assessments prior to conception, diligent oral health management throughout pregnancy, and meticulous oral hygiene practices during infancy. Effective strategies should be seamlessly integrated across the life course, encompassing preconception oral assessments, systematic dental care during pregnancy, and routine infant oral hygiene. Collaborative efforts among pediatric dentists, maternal and child health workers, and obstetricians are crucial to improving outcomes and fostering clinical research, contributing to evidence-based health management strategies.

## Introduction

Maternal and child oral health, a pivotal yet often overlooked component of maternal and infant health, has historically received insufficient recognition.^1,2^ Emerging evidence underscores the significant correlation between maternal oral health challenges during pregnancy,^1,3^ particularly periodontal diseases, and adverse outcomes such as preterm birth, low birth weight, and gestational hypertension.^4^ Oral health issues in pregnant women not only affect their own health but can also influence fetal development through various mechanisms, potentially heightening the offspring’s susceptibility to oral diseases.^5–7^ Pregnant women exhibit an incidence rate of dental caries 1.5–2 times higher than the general population,^8–10^ a vulnerability linked to hormonal changes, dietary shifts, and altered oral hygiene.^11,12^ This underscores the need for focused preventive dental care during pregnancy.^13,14^ They are also more likely to develop gingivitis, thus facing a greater risk to their oral health.^11,12^

Furthermore, oral health challenges during pregnancy not only intensify maternal complications during delivery but also exert lasting effects on fetal oral health, elevating the likelihood of future oral pathologies.^15–17^ Therefore, establishing standardized treatment protocols for maternal and child oral health is essential.^18^ By creating uniform guidelines, these protocols can provide guidance for the management of oral health in women and children, promote early intervention, and reduce the pregnancy complications caused by oral problems.^19^ This consensus, developed through an iterative modified Delphi process involving multidisciplinary experts, aims to guide oral health professionally in the prevention and management of oral diseases in women and children, while advocating for further research into the interconnected impacts of maternal and childhood oral health on overall maternal and infant well-being.

## Fetal Development and Oral Health of Pregnant Woman

### Maxillofacial development

Maxillofacial development originates primarily from the first pharyngeal arch, with the merging or fusion of the facial prominences occurring between the 6^th^ and 7^th^ week of embryonic development. The facial structures begin to take form during the 7^th^ to 8^th^ week, at which point the maxillofacial morphology is initially established. Genes responsible for regulating the migration of mesenchymal cells and neural crest cells initiate facial development around day 28 of pregnancy.^20^ The maternal nutritional status, overall health, and microbiome directly affect fetal craniofacial development. Previous studies have shown that biological indicators of craniofacial morphology—including nutritional status^21^ growth factors and hormonal activity,^22^ physical stature,^23,24^ and bone maturation processes^25–28^—play a pivotal role in shaping craniofacial development. The development of the craniofacial complex is fundamental for achieving harmonious relationships among the teeth, jawbones, and associated facial structures integral to occlusion. Disorders in craniofacial structure development can result in malocclusion, necessitating orthodontic interventions and, in some cases, orthognathic surger.^29^ Poor oral health during pregnancy, particularly oral infections such as periodontal disease or caries, can lead to systemic inflammatory responses via bacterial translocation into the bloodstream, adversely affecting fetal craniofacial development and increasing the risk of anomalies such as dental development defects, facial deformities, and cleft lip and palate.^30^

### Tooth development

Tooth development begins during fetal life, with the formation of the primary tooth buds occurring between the 6^th^ and 8^th^ weeks of embryonic development. This process lasts approximately 10 weeks. The initiation of permanent tooth development begins around the 20^th^ week of pregnancy, while mineralization starts postnatally and may continue until early adulthood.^31–33^ The development of the permanent molar tooth buds begins at the 20^th^ week of pregnancy (for the first permanent molar) and continues until the age of 5 (for the third permanent molar). Disruptions in this process can lead to various tooth development abnormalities. Maternal nutritional status, particularly the consumption of critical elements like calcium, phosphorus, and vitamin D, exerts a direct influence on the fetal tooth mineralization process.^34,35^ Prenatal exposure to viruses, pharmaceuticals, environmental contaminants, and ionizing radiation during early pregnancy can adversely affect odontogenesis, particularly altering the number, size, and morphology of developing teeth.^36^ Maternal deficiencies in protein, vitamin A, D, or minerals such as calcium, phosphorus, and iron can directly affect tooth development, resulting in structural abnormalities in enamel or dentin.^37^ Maternal systemic diseases also influence fetal tooth development. Seminal studies suggest a potential association between maternal diabetes and developmental anomalies in infant primary dentition.^38–40^ Maternal malnutrition during pregnancy is a significant contributor to low birth weight in offspring and has been strongly linked to the occurrence of enamel hypoplasia. Enamel hypoplasia is significantly more prevalent in the primary dentition of low birth weight infants than in those with normal birth weight, indicating a link between birth weight and tooth enamel development.^41^

### Oral microbiota and fetal growth and development

#### Maternal and infant microbiota and oral health

Maternal microbiota transmission is essential for the establishment of the infant’s oral microbiota, influencing early microbial composition and potentially impacting long-term oral health outcomes.

#### Placental microbiome and oral microbiota linkage

A study by the Aagaard team^42^ demonstrated that the placental microbiome mirrors the bacterial species prevalent in the maternal oral cavity, suggesting that oral microbiota may translocate via the maternal bloodstream to the placenta, potentially influencing fetal development.

#### Maternal oral health impact on bacterial transfer

Maternal oral health has been shown to influence the transference of bacteria from mother to child.^43,44^ Suboptimal maternal oral health is associated with elevated microbial loads and increased frequency of microbial transfer. Maternal health status, dietary patterns, and antibiotic usage modulate: metabolites generated by the microbiota and transference of microbiota or metabolites to the fetus.^44^

#### Fetal exposure and microbiome development

Maternal health status, dietary patterns, and antibiotic usage can modulate the metabolites generated by the microbiota and their transference to the fetus; through the placenta, the fetus is exposed to maternal microbiota and associated metabolites, which contribute to the formation of the fetal microbiome.^45,46^

#### Paternal microbiome contribution

Suboptimal maternal oral health is commonly linked to elevated microbial loads and increased frequency of microbial transfer. The paternal microbiome also plays a role, with the father not only being a genetic donor but also influencing the health of the offspring through their own microbiome, contributing to a mother-father-fetus microbiome interaction system.^47^

#### Impact of birth mode on microbial succession

The shift from prenatal to postnatal life requires several critical adjustments in neonatal respiratory, metabolic, and immune systems, alongside initial exposure to antibiotics and maternal microbiota. The mode of delivery significantly impacts these adaptations, playing a significant role in shaping the infant’s oral microbiota.^48^ At birth, vaginal delivery directly exposes the neonate to the maternal vaginal and rectal microbiota, while cesarean delivery introduces microorganisms from the maternal skin and hospital environment, shaping the infant’s early microbial composition.

#### Early microbial composition differences

Studies have shown that within the first two days post-delivery, the salivary bacterial count in cesarean-born infants is significantly lower, with lower detection rates of certain oral taxa such as *Actinomyces viscosus*, *Bifidobacterium dentium*, and *Streptococcus sanguinis*.^49^ Post-cesarean delivery, certain microbial communities show delayed colonization and lower diversity.^50^ Infants born via vaginal delivery exhibit higher oral microbiota richness, while those born via cesarean delivery tend to have a more homogeneous microbiota, which may impact immune system development.

#### Confounding effects of antibiotic exposure

However, given that antibiotics are routinely administered during cesarean delivery,^51^ evidence indicates that the infant’s microbiota is influenced by both the mode of delivery and antibiotic exposure. Infants exposed to antibiotics at birth show a reduced similarity between their oral microbiota and that of the mother, with a higher proportion of *Firmicutes*.^52^

#### Impact on breast milk composition

Interestingly, the mode of delivery also influences maternal breast milk, which serves as the primary substrate for the infant’s microbiota. After vaginal delivery, colostrum has a significantly higher total protein content than in cesarean-delivered infants, and the microbial composition of colostrum is similarly influenced by the mode of delivery.^53,54^

#### Impact of breastfeeding on microbial succession

Breastfeeding is vital for the proper development of the infant’s oral microbiota. Breast milk not only contains immune factors but is also rich in beneficial prebiotics that support the establishment of a healthy oral microbiome in newborns.

##### Early-Life Microbial Similarity Across Body Sites

Shortly after birth, the microbiota of the infant’s gut, mouth, nose, and skin is similar.^55–57^ By the age of six months, the infant oral microbiota closely resembles that of the mother’s oral cavity, breast milk, and areolar microbiota.^57^

##### Maternal Microbial Transfer via Breastfeeding

Certain bacterial species are transferred from the mother to the infant through breastfeeding,^58^ with the maternal microbial community naturally colonizing the infant’s gastrointestinal tract.

##### Role of Human Milk Oligosaccharides (HMOs)

The primary carbohydrate in breast milk that babies cannot digest, human milk oligosaccharides, aid in nourishing the newly formed microbiota.^59^

##### Biochemical Synergy and Microbial Maturation

An exceptional biochemical synergy exists between the infant’s oral secretions and the components of breast milk, influencing both the oral and gut microbiota.^60^ Furthermore, breastfeeding is associated with a delay in microbial maturation, which corresponds with the findings in the infant gut microbiota.

##### Stability and Immune Protection

Infants who are breastfed tend to develop a more stable oral microbiota with better immune protection.^61,62^

##### Factors Influencing Microbial Colonization

Variations in microbial colonization between individuals are influenced by multiple factors, including maternal salivary microbial load, extent of close interactions, nutritional sources, and the receptivity of the child’s oral environment.^63^ Breast milk, along with its immune and bioactive factors, may reduce particular germs from colonizing the oral cavity.

## Maternal Systemic Health and Fetal Oral Health

### Maternal bacterial and viral infections

Infection with *Treponema pallidum* can result in “Hutchinson teeth” in the fetus.^64^ Pathogens from infectious diseases (e.g., upper respiratory infections, rubella, mumps, viral flu, etc.) can lead to enamel hypoplasia.

### Maternal systemic diseases

Maternal diabetes may lead to abnormal development of infant primary teeth.^39,65^ Oral diseases during pregnancy can cause opportunistic pathogens to enter the placenta via the bloodstream, increasing fetal susceptibility to dental caries or periodontal diseases.^66^

### Maternal drug use and exposure to chemicals

Maternal exposure to certain drugs and environmental chemicals during critical periods of pregnancy can lead to permanent dental anomalies in the offspring.

#### Tetracyclines

Bind to calcium ions in mineralizing tissues, resulting in yellow-brown banding discoloration of teeth (“tetracycline teeth”);^67,68^

#### Thalidomide

Associated with supernumerary teeth or congenital tooth agenesis due to its teratogenic effects on craniofacial development.^69^

#### Antiepileptic drugs

Impair ameloblast function, leading to enamel hypoplasia with chalky white defects, especially in primary teeth.^70^

#### Co-exposure to lead, dioxins, or amoxicillin

These agents can exacerbate fluoride-related damage, worsening the extent of enamel defects.^71–73^

#### Excess Vitamin A or retinoic acid

Suppresses bone formation, stimulates osteoclastic activity, and induces enamel hypomineralization by disrupting odontogenesis.^74,75^

#### Excessive fluoride

Leads to dental fluorosis, characterized by mottled, discolored, or pitted enamel depending on severity.^76^

#### Bisphenol A (BPA)

Perinatal exposure has been linked to enamel developmental defects, likely through endocrine-disrupting mechanisms affecting ameloblast differentiation.^65,77^

#### Pesticide exposure

Prenatal exposure to pesticides(eg. Organophosphates)may interfere with cellular signaling pathways during tooth development, leading to enamel hypoplasia or malformed crowns.^78–81^

#### Heavy metal exposure

These toxicants can impair the function of ameloblasts and odontoblasts, resulting in delayed tooth eruption, enamel mineralization defects, and crown malformations.^82^

### Maternal malnutrition

Maternal malnutrition during pregnancy, leading to low birth weight, is associated with a significantly higher prevalence of enamel hypoplasia in primary teeth compared to infants with normal birth weight.^41^ It may also contribute to tooth morphological abnormalities such as microdontia and altered crown shapes due to disrupted odontogenesis.^83,84^

## Oral Health Management and Diseases during Pregnancy

### Periodontal diseases

The uniqueness of oral health challenges during pregnancy is primarily driven by hormonal fluctuations. Gingivitis is among the most prevalent oral conditions during pregnancy, triggered by changes in pregnancy hormones, leading to vascular dilation. This allows oral microorganisms to enter the placenta directly or via maternal immune cells, where microbial cells or fragments may be retained in the placental tissue, presenting antigens to the fetal immune system.^85–89^ Elevated progesterone levels during pregnancy alter the oral microbial environment, promoting the growth of Prevotella intermedia, a key pathogenic bacterium in periodontal disease.^90^ Some pregnant women may develop localized gum swelling, known as pregnancy granuloma or pyogenic granuloma, which occurs in about 5% of women. This condition may be due to higher hormone levels or a stronger inflammatory response to bacteria or trauma, resulting in increased angiogenesis. A systematic review found that maternal periodontitis is linked to a 1.6-fold increased risk of preterm birth, a 1.7-fold increased risk of low birth weight infants, and a 2.2-fold increased risk of preeclampsia. The combined likelihood of preterm birth and low birth weight is elevated by 3.4 times.^4^

### Pulpal diseases

Frequent vomiting during early pregnancy can increase the oral acidic environment, thereby raising the risk of dental caries. Over 43% of expectant mothers experience oral health issues, such as dental infections and discomfort, according to a new study.^91^ Most odontogenic infections may quickly develop into deep infections that threaten the oral-pharyngeal airway.^92^ Additionally, odontogenic infections often present with oral pain and swelling.^93^ Drug abuse in place of appropriate dental care can be detrimental to both the fetus and the pregnant woman. Therefore, prompt treatment of odontogenic infections during pregnancy is essential to minimize risks.^94^

### Pericoronitis of the third molars

Pericoronitis of the third molars is more prevalent during pregnancy, causing significant discomfort for the pregnant woman and potentially leading to anemia and malnutrition.^95^ The third molar may be partially or fully covered by a gingival flap, which forms a deep pocket between the gum and tooth, where food and bacteria can easily become trapped.^96^ When systemic immunity declines, pericoronitis becomes more likely, causing difficulties with eating, chewing, swallowing, and limited mouth opening.^97^ In severe cases, it can lead to infections of adjacent tissues, organs, or spaces. The risk of pericoronitis is increased by hormonal changes during pregnancy, poor dental hygiene, and rapid fetal growth in the latter stages of pregnancy.^66^

### Timing of treatment

- **Early Pregnancy:** The most crucial time for fetal growth is during the first trimester. If acute dental pain occurs in pregnant patients, symptomatic emergency management can be performed, such as removing inflamed pulp or draining pus to alleviate pain.
- **Mid-Pregnancy**: The safest time to have surgery or dental work done is during the second trimester. Emotional stability, reduced stress, and lower anxiety levels make it an ideal time for selective and necessary dental treatments, including professional plaque removal, periodontal surgery, tooth extractions, and root canal therapy.^98–102^
- **Late Pregnancy**: In case of dental pain, emergency treatment can be performed; however, it is advised to postpone treatment if possible, until after delivery.^103,104^ The American Dental Association underscores the importance of timely dental care during pregnancy.Controlling and treating odontogenic infections is essential for a healthy pregnancy.^105^ It is crucial to note that infections pose risks to both the mother and the fetus.

### Oral radiographic examinations and medication during pregnancy

#### Radiographic examinations

Guidelines from the United States (2003) and the European Union (2004) indicate that indications for X-ray examinations are the same for pregnant patients as for the general population.^106,107^ For elective dental procedures, radiographic examinations should be postponed until after pregnancy. However, in urgent cases, the lowest possible radiation dose should be used to meet diagnostic needs.^108^ Although evidence suggests that the radiation dose received by the fetus during oral X-rays is minimal and can be considered negligible,^106,107,109–111^ due to psychological concerns, X-ray examinations should be avoided during pregnancy whenever possible.^110,112^ It should be emphasized that the most appropriate imaging examination should be selected based on the diagnostic and therapeutic needs of the patient, adhering to the ALADAIP principle.^113^

In pediatric patients, radiographic examinations play a key role in early diagnosis and treatment planning for malocclusion. According to the latest expert consensus,^114^ age-specific imaging is recommended.^115^ Panoramic radiographs are generally preferred for initial screening, while lateral cephalometric radiographs are recommended for detailed assessment in early orthodontic planning. Cone-beam computed tomography (CBCT) should be reserved for cases where additional three-dimensional evaluation is necessary.^116^

#### Local anesthesia

Local anesthetics are commonly used in dental procedures to control pain, often combined with epinephrine to increase anesthetic depth and duration, improving pain relief. However, intravenous epinephrine administration can cause uterine artery constriction, leading to reduced uterine blood flow and potentially affecting fetal oxygen and nutrient supply. Studies indicate that using a 100 000 concentration of epinephrine is considered safe for healthy pregnant women in dental treatments.^117,118^ When administering local anesthetics containing epinephrine or other vasoconstrictors, the lowest possible concentration should be employed, and aspiration techniques should be used to avoid accidental intravascular injection.^119^ Current studies affirm that lidocaine is safe for use as a local anesthetic in pregnant women, with minimal risk to maternal and fetal health when administered properly.^105^

#### Infection-control medications

Irrigants such as sodium hypochlorite and root canal filling materials are not considered harmful to the fetus.^92^ In cases of severe odontogenic infections, antibiotics such as amoxicillin and metronidazole may be used. established by the American Dental Association and obstetricians, certain antibiotics are permissible during pregnancy and can be used safely. Some antibiotics may have teratogenic effects during pregnancy, and evidence suggests that amoxicillin use during pregnancy may lead to congenital defects such as cleft lip/palate and enamel hypomineralization.^120–122^ The combination of amoxicillin and clavulanic acid has been shown to be effective in managing severe odontogenic infections, with no evidence indicating an elevated risk of fetal or congenital abnormalities.^105^ However, it has been observed that the use of this combination during the later stages of pregnancy may elevate the risk of necrotizing enterocolitis in newborns.^123^ Therefore, its use should be avoided during the third trimester.^105^ Metronidazole, commonly used to target anaerobic bacteria, is often combined with penicillin to treat odontogenic infections. Research indicates that metronidazole use during pregnancy is not significantly linked to fetal defects or adverse pregnancy outcomes.^124–129^ In contrast, tetracycline antibiotics have been shown to cause staining and discoloration of permanent teeth,^67,68^ and it is recommended to avoid their use during pregnancy. Medications used during breastfeeding can severely impact infant health. Therefore, antibiotic use for odontogenic infections should be considered carefully during pregnancy and breastfeeding, with consultation from obstetricians.

### Nutritional intake and oral health during pregnancy

Research indicates that adequate intake of calcium, phosphorus, and vitamin D intake is crucial for the healthy formation and mineralization of teeth and jawbones.

#### Consequences of Vitamin D deficiency

Vitamin D deficiency impacts the oral cavity, leading to enamel hypoplasia, dentin changes, and ectodermal dysplasia, particularly affecting primary teeth and potentially resulting in defects in permanent dentition. This condition affects both esthetics and increases sensitivity, along with elevating the risk of caries, and may lead to reduced bone mineral density, resulting in jawbone resorption.^130^

#### Role in fetal dental development

Fetal tooth development depends on vitamin D, and maternal vitamin D during pregnancy is essential for proper tooth mineralization and the development of healthy dental structures in the fetus.^131^ Studies indicate that optimal vitamin D levels are linked to a reduced incidence of dental caries,^132–134^ with supplementation preventing both its onset and progression.^135–138^ High levels supplementation during pregnancy may mitigate the risk of enamel defects in newborns.^136^

#### Critical periods in tooth development

Early pregnancy (1–3 months) sees primary tooth bud initiation, mid-pregnancy (4–6 months) involves primary tooth mineralization, and late pregnancy (7–9 months) continues primary tooth development/mineralization and permanent tooth bud formation. Pregnant women should ensure sufficient intake of high-quality proteins, calcium, phosphorus, vitamin D, and essential minerals to support the formation of teeth and jawbones.^135,136^

### Oral hygiene care during pregnancy

The incidence of dental caries may increase during pregnancy, owing not only to physiological changes but also to heightened cravings for sugary foods, challenges in maintaining adequate oral hygiene, and pregnancy-related nausea and vomiting.^139^ Additionally, dental caries can lead to periapical periodontitis, which, akin to periodontal disease, may be linked to adverse pregnancy outcomes.^140^ Due to the heightened vulnerability to oral health issues during pregnancy, preventive strategies are essential to manage the increased risk of conditions like gingivitis and periodontitis. Pregnant women should maintain good oral hygiene, reduce sugar intake, avoid carbonated drinks and acidic foods, and undergo regular dental check-ups with timely plaque removal. Maintaining optimal oral hygiene during pregnancy aids in reducing the transmission of cariogenic bacteria, giving high-risk mothers the opportunity to implement preventive measures against caries transmission to their children.^141^

## Preconception Oral Health Management

A comprehensive oral examination before conception is crucial for improving oral health during pregnancy. Studies have shown that elevated progesterone and estrogen during pregnancy increase vascularization in the periodontium, alter collagen production, and enhance biofilm accumulation, potentially worsening existing gum inflammation or periodontal disease.^142,143^ A preconception oral check-up can effectively identify potential oral health issues, including dental caries, periodontal disease, and impacted third molars. It is recommended that women undergo an oral examination before conception to assess the eruption status of the third molars and, if necessary, extract impacted third molars that meet the indications for removal. Timely treatment of these oral issues can reduce the risk of complications during pregnancy. Performing an oral examination six months before conception and addressing any identified oral issues, such as caries treatment and periodontal cleaning, can effectively prevent oral health problems during pregnancy. The American Academy of Periodontology, the American Dental Association, and the European Federation of Periodontology emphasize the importance of oral health interventions prior to pregnancy to reduce the risk of adverse pregnancy outcomes.^98–100^ Oral health is intricately linked to the oral microbiome. While the maternal microbiome is not directly transferred to the fetus, it plays a vital role in priming the fetal immune system for post-birth microbial exposure, through modulation and training. Therefore, maintaining optimal maternal oral health and a balanced oral microbiome is essential for the child’s overall health and well-being.^47^ Oral health management during the preconception period is vital for maintaining a proper microbial balance and reducing the risk of bacterial infections.

## Oral Health Management in Infancy and Early Childhood

The eruption of the first primary teeth creates stable surfaces promoting microbial attachment and proliferation, increasing diversity.^144^ From the first tooth eruption, parents should be educated about caries risk. Parents should clean the infant’s oral cavity nightly from birth to remove milk residue and facilitate learning proper hygiene habits. Research demonstrates prolonged breastfeeding is associated with an elevated risk of early childhood caries. If breastfeeding is frequent, especially nightly, without adequate hygiene, multiple caries may develop, hindering growth.146 Some studies^145^ suggest infant formula and bottle-feeding are linked to increased risks of mouth breathing, malocclusions, and caries. Exclusive breastfeeding for the first six months is linked to a reduced risk of malocclusion (e.g., open bite, crossbite)^146,147^ and lower incidence of developmental abnormalities in primary teeth.150 Children exclusively breastfed over six months exhibit greater intercanine and intermolar width.^148^ The World Health Organization (WHO) advocates for exclusive breastfeeding during the first six months.^149,150^

Sucking is an innate reflex. The muscle activity in breastfeeding helps develop oral motor skills,^151^ enhancing tone and ensuring optimal function.^152^ Habits like pacifier use introduce unique stimuli affecting development.^153,154^ Early pacifier use may interfere with suckling and cause nipple confusion.^155^ Furthermore, pacifier use is associated with higher oral candidiasis incidence.^156,157^ Non-nutritive sucking behaviors correlate with primary dentition malocclusion.^153,155^ If breastfeeding lasts less than six months, thumb-sucking frequency may increase.^158^

## Conclusion

**Fig. 1:**
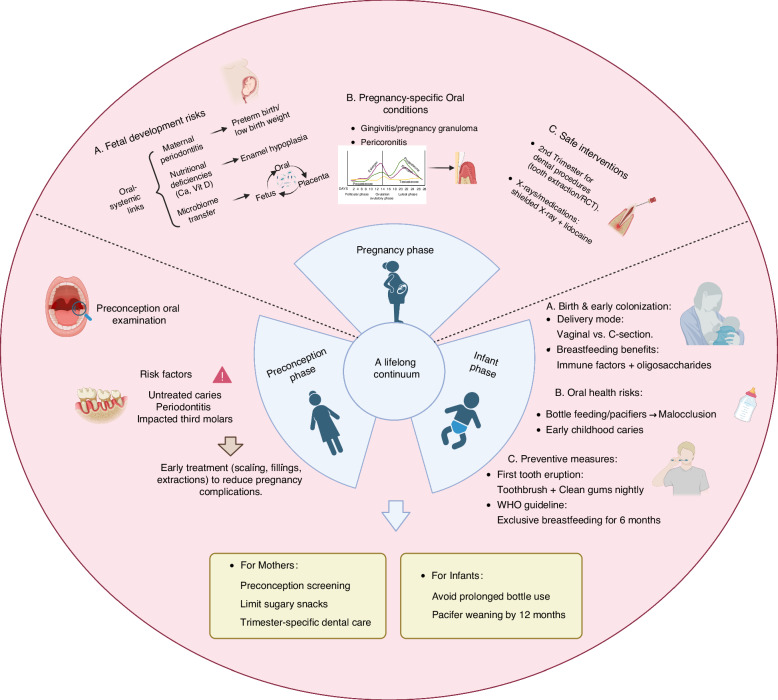
This schematic illustrates the critical pathways linking maternal oral health to fetal development and infant oral health outcomes, as established in this expert consensus. Key components include: Preconception Phase (Left Panel):Highlights the importance of pre-pregnancy oral screening (e.g., caries, periodontitis) and early interventions to mitigate gestational risks. Pregnancy Phase (Center Panel): Demonstrates bidirectional relationships between maternal oral diseases (gingivitis, periodontitis, caries) and adverse pregnancy outcomes (preterm birth, low birth weight), mediated by systemic inflammation and microbial translocation.Identifies the second trimester as the optimal period for dental treatments, with cautionary notes on radiation/medication safety.Infant Phase (Right Panel): Shows how delivery mode (vaginal vs. cesarean) and feeding practices (breastfeeding vs. formula) shape the infant’s oral microbiome and occlusal development, influencing lifelong caries and malocclusion risks. The image was created using BioRender.com (accessed on 6 April 2025). RCT, root canal therapy

The management of maternal and infant oral health is a continuous, lifelong process that begins before conception, extends throughout pregnancy, and persists into infancy and early childhood. As illustrated in Fig. 1 and Table 1, this continuum underscores the profound interconnections between maternal oral health, fetal development, and long-term infant well-being. Timely intervention and scientific management are essential at each stage. Maternal oral health during pregnancy has a direct impact on the overall well-being of both the mother and the child. Therefore, both dental professionals and obstetricians should strengthen oral health education and prevention during pregnancy. Future research should further investigate the long-term effects of oral health on maternal and child health, and rigorously validate existing management protocols in clinical practice.

**Table 1:** Evidence-based Recommendations for Maternal and Child Oral Health

Domain	Key Findings/Recommendations	Evidence/Support	Significance
Maternal-fetal link	Periodontitis ↑ risk of PTB (1.6×), LBW (1.7×), preeclampsia (2.2×)^4^	Systematic review; microbial translocation → systemic inflammation	Mandates prenatal oral screening & intervention
Fetal development	- Maxillofacial: Oral infections/malnutrition disrupt W6-8 development → malocclusion^29,30^ - Tooth: Vit D/Ca/P deficiency → enamel hypoplasia ↑ in LBW infants^34,35,41^	Critical windows: tooth bud (W6-8), mineralization (prenatal-postnatal)^31–33^	Highlights developmental vulnerability to nutritional deficits
Microbiome dynamics	Maternal oral microbiota transfers via placenta.^42^ and vertical transmission^43,44^ Vaginal delivery → richer infant microbiota vs. C-section^48–50^	Placental microbiome mirrors oral cavity^42^; breastfeeding stabilizes via HMOs/immune factors^59–62^	Supports pre-conception oral hygiene & vaginal delivery
Clinical management	Trimester-specific: - T1: Emergencies only - T2: Optimal for treatment - T3: Defer elective care Imaging: ALADAIP principle^107–114^ Anesthesia: Lidocaine + 1:10^5^ epinephrine safe^118–120^	Hormonal changes ↑ *P. intermedia* growth^91^; pericoronitis ↑ malnutrition risk^96–98^	Validates safety protocols for essential interventions
Interventions	Nutrition: Vit D supplementation ↓ caries risk & enamel defects^131–139^ (Grade B) Preconception: Screening ↓ pregnancy complications^99–101,143,144^	High-dose prenatal Vit D ↓ enamel defects^137^; maternal microbiome primes fetal immunity^47^	Guides supplementation & early care coordination
Infant health	Exclusive breastfeeding ≥6 months ↓ malocclusion^147–149^ Pacifier use ↑ candidiasis/malocclusion^154–158^	Breastfeeding ↑ intercanine width150; nocturnal feeding + poor hygiene → ECC^150^	Reinforces WHO breastfeeding guidelines & early oral hygiene^151^

*PTB* Preterm birth, *LBW* Low birth weight, *HMOs* Human milk oligosaccharides, *ECC* Early childhood caries, *WHO* World Health Organization, *T1/T2/T3* Trimester 1/2/3
